# Environmental analysis of returnable packaging systems in different eCommerce business and packaging management models

**DOI:** 10.1111/jiec.13537

**Published:** 2024-08-13

**Authors:** Jonghun Park, Zuha Waqar, William Ralph Snyder

**Affiliations:** 1 The Creative School, Toronto Metropolitan University, Toronto, Ontario Canada; 2 Environmental Applied Science and Management, Toronto Metropolitan University, Toronto, Ontario Canada

**Keywords:** closed-loop supply chains, eCommerce, life cycle assessment, packaging, reuse, sustainability

## Abstract

There is growing environmental concern regarding the increasing quantity of packages in retail eCommerce. This study investigated the environmental impact of two returnable packaging formats, performing life cycle assessment (LCA) case studies based on the Canadian apparel eCommerce market. In case study 1, the brand owner sold and shipped its products to final consumers using an expendable mailer and a returnable mailer that was managed and supplied via the centralized model. In case study 2, the brand owner rented its products to final consumers and shipped them using an expendable corrugated paperboard box and a returnable box that was managed and supplied via the decentralized model. Comparative, contribution, and sensitivity analyses were conducted to analyze and compare the environmental performance of these packaging options. For case study 1, the LCA revealed that the returnable mailer had greater impact than the expandable mailer in 9 of the 10 environmental impact categories, even if the returnable mailer was reused for 40 cycles and the final consumer was in the same city as the brand owner; this was primarily due to the length of transportation. For case study 2, the returnable box had smaller environmental impact than the expendable corrugated paperboard box in 6 of the 10 environmental impact categories, even though the brand owner shipped packages to final consumers a cumulative distance of 9000 km from its starting location. The overall results imply that the environmental burden of returnable packaging is primarily affected by total trip distance and the number of reuses.

**Supplementary Information:**

The online version of this article (doi:10.1111/jiec.13537) contains supplementary material, which is available to authorized users.

## INTRODUCTION

There are growing environmental concerns regarding the booming number of packages used in the eCommerce market (Baskoro, 2020). In recent years, there have been active discussions and new initiatives and guidelines introduced in the eCommerce sector to reduce the environmental impact of the growing number of packages in global eCommerce supply chains. Although sustainable eCommerce packaging developments have often focused on packaging materials and cubic reductions, with the use of post-consumer materials, and alternative packaging material development such as biodegradable polymers (Escursell et al., 2021), there has been an increasing attention toward reusable or returnable packaging systems for sustainable eCommerce (Greenwood et al., 2021).

In terms of the circular economy and its 4R framework (Reduce, Reuse, Recycle, and Recover), returnable packaging has been considered as one of the most sustainable packaging options in the packaging industry (Golding, 1999; Macarthur, 2013). While the terms “reusable” and “returnable” are typically used interchangeably in the context of packaging (Soroka, 2007), returnable packaging is a feasible option for supply chains that have a reverse logistics system (Coelho et al., 2020; Greenwood et al., 2021). As an alternative to single-use packaging, returnable packaging has been used in many traditional industrial sectors where the reverse logistics system is applicable such as fresh produce, automotives, and beverages (Accorsi et al., 2020). While economic benefits such as packaging material cost reduction are the primary motivation for the use of a returnable packaging system (Mollenkopf et al., 2005; Twede & Clarke, 2008), many life cycle assessment (LCA) studies have also reported that returnable packaging generated lower environmental impacts than single-use packages in various business-to-consumer (B2C) and business-to-business (B2B) traditional retail channels (Goellner & Sparrow, 2014; Koskela et al., 2014; Lee & Xu, 2004; Levi et al., 2011; Tua et al., 2019).

Despite the fact that it has not been a common option in retail eCommerce (Coelho et al., 2020), there is an increasing number of returnable packaging suppliers focusing on retail eCommerce such as RePack, Returnity, Zalando, LimeLoop, Otto, and LivingPackets. Notably, returnable packages are increasingly used by apparel brand owners who ship their products to consumers through parcel delivery services (Jestratijevic et al., 2022). Depending on the associated supply chains, there are two types of returnable packaging models in retail eCommerce: the centralized system and the decentralized system. In the centralized system, the package delivered to the final consumer is returned to a maintenance facility of the third-party returnable packaging supplier for cleaning and maintenance and is supplied again to a brand owner. However, in the decentralized system, the used package is returned back to the brand owner's location and cleaned and maintained by the brand owner or its local cleaning/maintenance partner for the next use (Hugill et al., 2021).

Recent LCA studies have compared the environmental impacts between returnable packaging systems and expendable packaging systems. Hugill et al. (2021) compared the environmental impact of returnable shipping bags to two expendable packaging options (expendable mailer and corrugated paperboard box) based on fast-track LCA results. The fast-track LCA was conducted based on European supply chains with one impact category, greenhouse gas (GHG) emissions. The study reported that the returnable mailer had lower environmental impact for GHG emissions than the expendable mailer if the returnable mailer travelled up to 4000 km per cycle and was reused for 30 cycles. The returnable mailer had significantly less GHG emissions than the corrugated paperboard box. It also found that the decentralized reusable model generated 53% lower GHG emissions than the centralized reusable model due to the greater transportation distance of the centralized reusable model. Zimmerman and Bliken (2020) also conducted an LCA based on apparel eCommerce in Germany to compare the GHG emissions (CO_2eq_ emissions) of a returnable plastic box, a returnable mailer, and an expendable mailer. The study reported that the returnable mailer generated the same environmental impacts as the expendable mailer if the returnable mailer was used for eight cycles and travelled up to approximately 4500 km per cycle. A recent review paper (Escursell et al., 2021) discussed current and alternative eCommerce packaging options in terms of environmental sustainability. The article pointed out that more LCA studies are required to guide stakeholders to the proper choice of sustainable packaging options for e-commerce.

While the previous studies found valuable results regarding apparel eCommerce, they also had some limitations. First, they were only conducted for a single environmental impact category (i.e., global warming potential). As there are different types of environmental impact categories that need to be reviewed for an environmental comparison, the LCA results based on a single impact category could not represent the holistic environmental performance of those packaging options. Also, the previous LCA studies were conducted based on the geographical scope of Europe. There is no LCA study analyzing the environmental performance of returnable packaging based on Canadian eCommerce. A different geographical scope could result in different results. Also, the previous studies only focused on selling business models.

On the other hand, rental subscription services have become more common in the eCommerce market. While some traditional brands started offering product rental services, there have been an increasing number of new companies that focus on rental subscription services. There is a lack of knowledge regarding the environmental performance of returnable packaging on a rental business model. The rental business model could differ from the typical selling model in terms of consumer engagement and return rate due to the models having different levels of obligation and financial penalty policies regarding non-return; these differences can then affect the environmental burden of associated returnable packaging.

The objective of this study is to investigate the environmental sustainability of two different returnable packages used for apparel eCommerce in Canada, including different eCommerce business models such as a selling model and a rental subscription model. The intended audience of this study includes eCommerce businesses, packaging designers, sustainability consultants, policymakers, and environmentally conscious consumers in Canada. The results will provide valuable insights into the environmental impacts of returnable packaging systems, helping businesses and designers make informed decisions about sustainable packaging solutions. Policymakers can use the findings to develop regulations that promote eco-friendly practices, while consumers will be better equipped to choose sustainable options in their online shopping. The findings of this study will fill the missing gaps on the environmental performance of returnable packaging systems in Canadian eCommerce supply chains and guide consumers and corporations to sustainable packaging system choices.

## METHODS

LCAs were performed on two case studies to investigate the environmental impacts of two returnable packaging formats in different eCommerce business models (i.e., a selling and buying model and a rental model) and different returnable packaging management models (i.e., a centralized model and a decentralized model). The LCAs complied with ISO 14040 (ISO, 2006a) to 14044 (ISO, 2006b) standards, consisting of four stages: goal and scope definition, inventory analysis, impact analysis, and interpretation. The foreground data were collected from the brand owners and returnable packaging suppliers in 2021. LCA software SimaPro v9.0 was used to analyze the data. The primary LCA audience included brand owners selling or renting non-perishable and non-fragile items through e-commerce in North America.

### Scope of the LCAs

Case study 1 compared a returnable mailer (R1) to an expendable mailer (S1) used to ship a skirt from brand owner A in Anchorage, Alaska (AK), USA to its customer in Vancouver, British Columbia (BC), Canada. The functional unit used for the LCA in case study 1 was packaging to ship 10,000 skirts individually to the final consumers. Case study 2 compared a returnable box (R2) to an expendable box (S2) used to rent a bundle of baby clothes (24 items per bundle) from brand owner B in Vancouver, British Columbia (BC), Canada, to its customer in Toronto, Ontario (ON), Canada. The functional unit used for the LCA in case study 2 was packaging used to ship 10,000 bundles of baby clothes individually to the final consumers who returned the used clothes to the brand owner after use for the next rental. The scope of the LCAs was cradle-to-grave, which included the packaging life cycle from raw material extraction to disposal phase.

### Material flow descriptions and system boundaries

The description of the packaging scenarios and packaging specifications assessed in the two case studies are listed in Table Table 1.

**TABLE 1:** Description of the case study packaging scenarios.

	Case study 1	Case study 2
**Product type**	Skirt	Baby clothing
**Brand owners**	Brand owner A (Anchorage, AK, USA)	Brand owner B (Vancouver, BC, Canada)
**Business model**	• Selling model • Non-subscription based • Non-membership based	• Rental model • Subscription based • Membership based
**Customer's location**	Vancouver, BC, Canada	Toronto, ON, Canada
**Returnable packaging scenarios**	• Centralized model • Reusable mailer primarily made of polypropylene (R1) ○ Weight: 132 g • Supplied by returnable packaging supplier A • Maintained by returnable packaging supplier A	• Decentralized model • Reusable box primarily made of polypropylene and polyester (R2) ○ Weight: 987 g • Supplied by returnable packaging supplier B • Maintained by brand owner B
**Returnable packaging scenarios: Distance per return cycle**	•Manufacturer to supplier: ○ 35,688 km (Ocean) ○ 1053 km (Truck) •Supplier to brand owner: ○ 4754 km (Truck) •Brand owner to customer ○ 3528 km (Truck) •Customer to supplier: ○ 1554 km (Truck) •Supplier to brand owner: ○ 4754 km (Truck)	•Manufacturer to brand owner: ○ 38,133 km (Ocean) ○ 17 km (Truck) •Brand owner to customer: ○ 4206 km (Truck) •Customer to brand owner: ○ 4206 km (Truck)
**Expendable packaging scenarios**	• Expendable mailer made of high-density polyethylene (S1) ○ Weight: 17 g	• Expendable corrugated paperboard box (S2) ○ Weight: 457 g
**Expendable packaging scenarios: Distance per one way shipment**	• Manufacturer to supplier: ○ 6968 km • Supplier to brand owner: ○ 15 km • Brand owner to customer: ○ 3528 km	• Manufacturer to supplier: ○ 9 km • Supplier to brand owner: ○ 15 km • Brand owner to customer: ○ 4206 km

#### Case study 1: Returnable mailer (R1) versus expendable mailer (S1)

**FIGURE 1:**
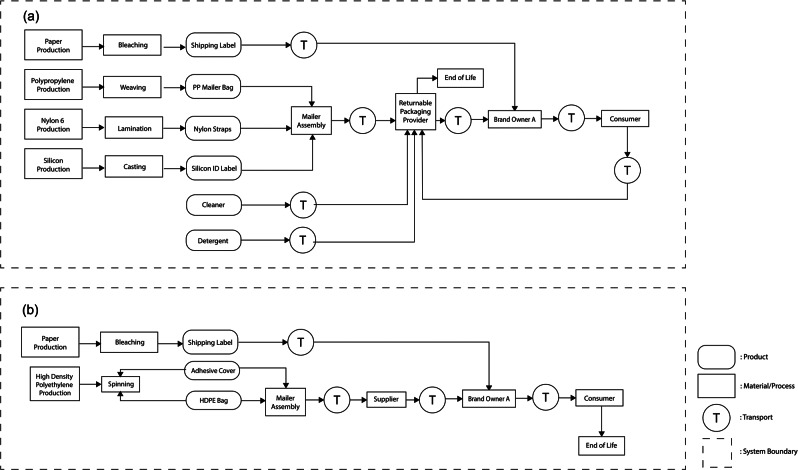
Systems boundaries of the two packaging scenarios analyzed in case study 1: returnable mailer (a) and expendable mailer (b).

Case study 1 compared the environmental impacts of a returnable mailer (R1) and an expendable mailer (S1) used to ship a skirt from brand owner A in Anchorage, AK, USA to its consumer in Vancouver, BC, Canada. The shipped product, skirts, are not included in the system boundaries of this study. Figure 1 provides further clarity on the system boundaries of the two packaging scenarios analyzed in case study 1.

##### Returnable mailer (R1)

The returnable mailer (R1) consisted of a body (woven PP), six straps (nylon fiber), an identification sticker (silicon), and a shipping label (bleached paper). After the mailers were manufactured in Shanghai, China, they were shipped to the returnable packaging service provider's facility in Salt Lake City, UT, USA, by truck and ocean freight. Then, the packages were delivered to brand owner A in Anchorage, AK, USA, by truck transportation. Once a consumer in Vancouver, BC, Canada, placed an order, the returnable mailer containing a skirt was delivered to the consumer. After receiving the product, the consumer returned the empty mailer to the returnable packaging service provider's facility in Salt Lake City, UT, USA, using ground transportation through Canada Post and the United States Postal Service. According to the returnable packaging service provider, the average loss rate of the returnable mailers was estimated to be approximately 30%. If mailers could be returned from the final consumers without a loss, the average reuse cycle per lifetime was 20 cycles. In the returnable packaging service provider's maintenance facility, the returned mailers were left to sit for 72 h to minimize COVID-19 risk and visually inspected to assess physical damages. Common damage included ink or glue contamination and tears. Damaged mailers were stored for recycling, and non-damaged mailers were wiped off with a microfiber cloth and a biodegradable cleaning spray. Any remaining label residue was removed using a knife and wiped with a damp cloth with detergent. No water was used during the cleaning process.

##### Expendable mailer (S1)

The expendable mailer (S1) was made of high-density polyethylene (HDPE) and an adhesive. The HDPE mailer was manufactured through an extrusion process in Charlotte, North Carolina (NC), USA. After manufacturing, the single-use mailer was shipped to brand owner A in Anchorage, AK, USA, by a single unit diesel truck. Brand owner A shipped a mailer containing a product to its consumer in Vancouver, BC, Canada. After the consumer received the product, the mailer was disposed of in a landfill (DuPont, 2017).

#### Case study 2: Returnable box (R2) versus expandable box (S2)

**FIGURE 2:**
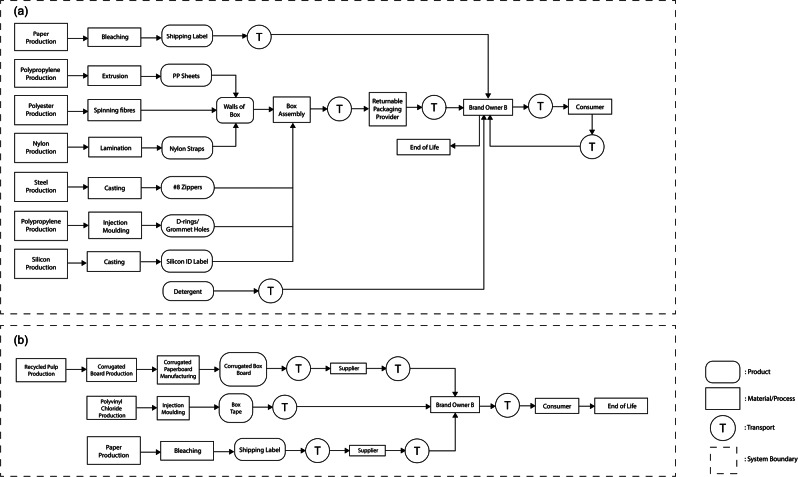
Systems boundaries of the two packaging scenarios analyzed in case study 2: returnable box (a) and expendable box (b).

Case study 2 compared the environmental impacts of two packaging scenarios (i.e., a returnable box and an expendable box) used to ship a bundle of baby clothes from brand owner B in Vancouver, BC, Canada to its consumer in Toronto, ON, Canada. The business model of brand owner B was a membership- and monthly subscription-based rental model. The brand owner shipped 24 pieces of baby clothes to its consumer monthly. After use, the consumer returned the clothes to the brand owners. The returned clothes were washed and conditioned to rent to another consumer. It is worth noting, that the shipped products, baby clothes, are not included in the boundaries of this LCA. Figure 2 describes the system boundaries of the two packaging scenarios analyzed in case study 2.

##### Returnable box (R2)

The returnable box (R2) consisted of a body, zippers, d-rings (polypropylene; PP), straps (nylon), an identification label (silicon), and a shipping label (bleached paper). The body (box walls) was made of polyester fabric and a PP sheet (4 mm thickness). The zipper consisted of two strips of nylon teeth and a metal slider. The components were manufactured and assembled as a box format in Xiamen, China. The manufactured returnable mailers were directly transported to brand owner B in Vancouver, BC, Canada, via container ships and trucks. To fulfill its customer's subscription, brand owner B shipped a bundle of baby clothes (24 items per bundle) monthly using a returnable box to its customers in Toronto, ON, Canada, via truck transportation. After using the clothes, the customer returned them to the brand owner using the returnable box through Canada Post's ground transportation.

According to brand owner B, the loss rate of the returnable box was zero primarily due to its rental, subscription-based, and membership-based business model. The member customers could not receive new products if they did not return the returnable box when they shipped the used clothes back to the brand owner. The returnable boxes were reused for an average of 40 cycles. Employees visually inspected all returned packages. Approximately 10% of packages required cleaning; common damages to the packages were permanent marker writing by Canada Post. The used packages were cleaned using a combination of detergent and water. After the returnable box reached the maximum number of reuses (40 cycles), it was sent to the landfill at the end-of-life stage.

##### Expendable box (S2)

The expendable corrugated box (S2) consisted of three components: a single-wall corrugated paperboard box (B-flute), box tape (polyvinyl chloride), and a shipping label (bleached paper). The corrugated paperboard box was manufactured in Richmond, BC, Canada and delivered to the brand owner B in Vancouver, BC, Canada. Using the expendable corrugated box (S2), the brand owner B shipped a bundle of 24 items of baby clothes to its customer in Toronto, ON, Canada, using Canada Post's ground transportation. At the end of the rental period, the customers returned the clothes to the brand owner using the same corrugated box, attaching a new shipping label and box tape. The corrugated box was disposed of via recycling after the brand owner received the box containing the used clothes.

### Life cycle inventory analysis

The life cycle of all packaging scenarios was divided into four phases: material production, intermediate processes, transportation, and disposal, to facilitate the understanding of the LCA results. The foreground data were obtained by the brand owners and their returnable packaging suppliers. Background data obtained from the literature were used when foreground data was unavailable. For both R1 and S1 analyzed in case study 1, data for material production and intermediate processes were obtained from US-EI 2.2 (Frischknecht et al., 2005). For the returnable and expendable boxes (R2 and S2) analyzed in case study 2, data from US-EI 2.2 and Ecoinvent were used for the material production and intermediate processes. The data for the transportation phases of all the packaging scenarios were obtained from USLCI (USLCI, 2012). The transportation distances were calculated utilizing Google Maps. Knowing the weights of packages and transport distances, the transportation data were converted to a ton-kilometer (tkm). Tables S1 and S2 show the life cycle inventory databases used for case study 1 returnable mailer and expendable mailer, and case study 2 returnable box and expendable box, respectively. The waste treatment scenarios of general solid wastes were assumed according to the government report published by Environment and Climate Change of Canada and US-EI 2.2. For the plastic materials, it was assumed that 86% of the total wastes went to landfills, 9% was recycled, 4% was incinerated, and 1% was litter. For the paper-based materials, it was assumed that 68% of the total paper-based materials were recycled, 12% were incinerated, 12% were landfilled, and 8% were litter. All the recycling, incineration, and landfill data were obtained from the US-EI 2.2 database.

### Life cycle impact assessment

The Tool for the Reduction and Assessment of Chemical and other Environmental Impacts (TRACI 2.1.), a mid-point life cycle impact assessment methodology, was used to calculate the environmental impacts generated from the input and output data (Bare, 2014). TRACI 2.1. was chosen as a life cycle impact assessment methodology because it was created based on the geographical and technological scope of North America, similar to the scope of the LCAs in this study. TRACI 2.1 includes 10 impact categories such as acidification, carcinogenicity, non-carcinogenicity, ecotoxicity, eutrophication, fossil fuel depletion, global warming, ozone depletion, respiratory effects, and smog production.

### Limitations and assumptions

The followings are assumptions and limitations included in this study:

- Although there was some space left to contain more items in the packages, it was assumed that a package contained one item or bundle of items for shipping at a time.
- There were slight cubes and size differences between comparable packages, as this study analyzed currently available and used packaging formats by the packaging suppliers and brand owners.
- The scope of this LCA did not include secondary and tertiary packaging components (e.g., overboxes, pallets, and stretch wrap) used to ship individual packaging components from production sites to brand owners and packaging suppliers.
- Although the protection levels of the varying types of packages analyzed in this study could be different, this study did not consider the different protection levels of the packages, as the packages only contain and ship non-fragile items (i.e., apparel).
- The end-of-life scenario for paper-based components referred to the US average (USEPA, 2020), because there is an absence of Canadian-based end-of-life scenarios for those components.
- This study assumed that the returnable packages not returned by the final consumers were disposed of according to the end-of-life scenario reported in Environment and Climate Change Canada (2019)
- It was assumed that the returnable packages were shipped to the same consumer's locations during their entire lifetimes.

## RESULTS

The environmental results were interpreted through contribution and comparative analyses performed at the level of characterization. In addition, sensitivity analyses were conducted to evaluate the robustness of the LCA results and their sensitivity to uncertain factors and assumptions.

### Case study 1: Returnable mailer (R1) versus expendable mailer (S1)

The results of the life cycle impact assessment of the returnable mailer (R1) and the expendable mailer (S1) are listed in Tables S3 and S4 in the supporting information, respectively.

**FIGURE 3:**
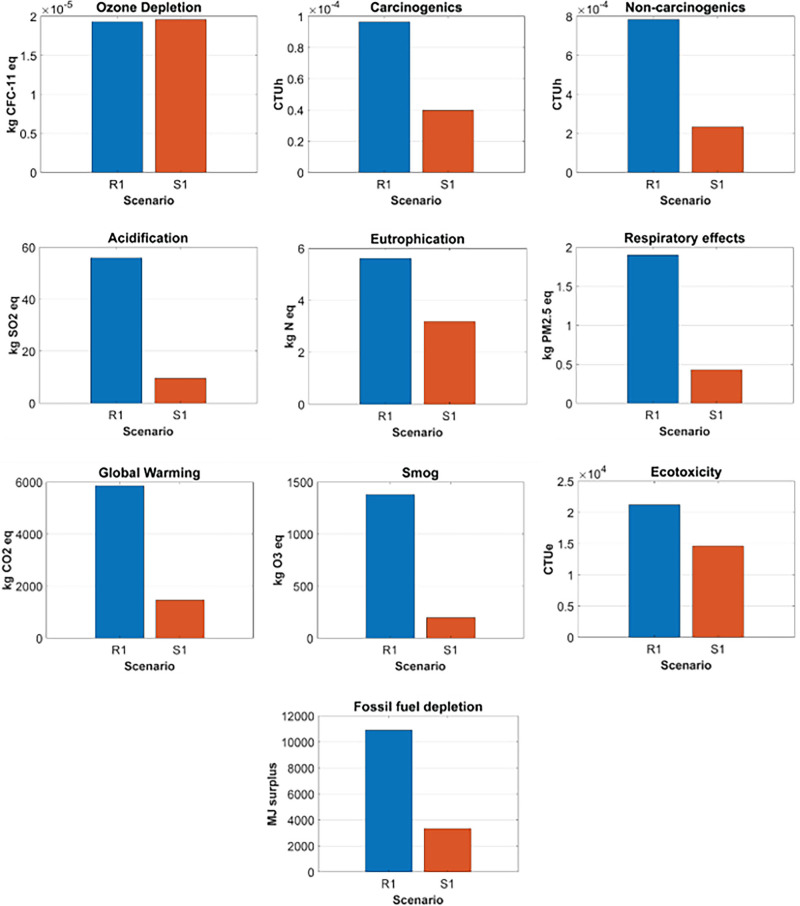
Comparative analysis of case study 1: R1 versus S1. Underlying data for this figure are available in Tables S3 and S4 of Supporting Information.

Figure 3 depicts the comparative analysis of R1 and S1. R1 had a greater environmental impact than S1 for all the impact categories, except ozone depletion, where R1 had approximately 1% lower environmental impact than S1. The most considerable difference was for smog, in which R1 generated 600% more environmental impact than S1. The second greatest difference was acidification, where R1 had 482% more environmental impact than S1. The primary reason for the difference between R1 and S1 was the longer transport distance of R1's supply chains.

**FIGURE 4:**
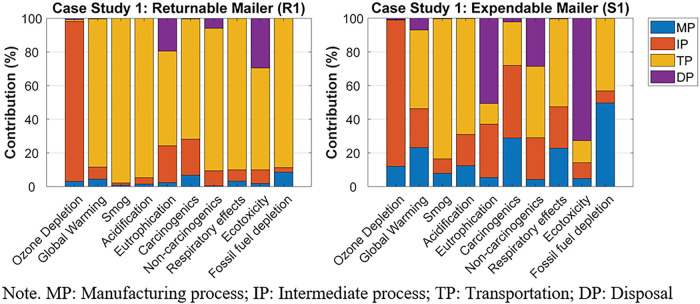
The normalized contribution analyses for case study 1's returnable mailer (R1) and expendable mailer (S1). Underlying data for this figure are available in Tables S3 and S4 of Supporting Information.

The normalized contribution of each life cycle stage's environmental impact, for R1 and S1, can be found in Figure 4. For R1, the transportation phase was the greatest contributor to the environmental impact categories, except ozone depletion. Specifically, the long-haul transport between the returnable packaging supplier in Salt Lake City, UT, USA, brand owner A in Anchorage, AK, USA, and the final consumer in Vancouver, BC, Canada, and the crude oil production and diesel refinery process for the gas consumed during the transports were the primary contributors to the nine environmental impact categories. The largest contributor to ozone depletion was the intermediate process, especially the weaving process of the PP yarns.

For S1, the transportation phase contributed the most to global warming, smog, acidification, non-carcinogenics, and respiratory effects. Especially, the long-haul transport between brand owner A and the final consumer, and the crude oil production and diesel refinery process for the gas consumed during the transports were the major factors contributing to the transportation phase. Material production was the greatest contributor to fossil fuel depletion due to the oil-derived raw materials used during HDPE production. The intermediate process (i.e., HDPE extrusion) contributed the most to ozone depletion and carcinogenics. The largest contributor to eutrophication and ecotoxicity was the disposal phase, specifically that the package ended up in the landfill.

The first sensitivity analysis for case study 1 was conducted to analyze the sensitivity of the LCA results depending on the number of reuses of R1. The original LCA results were based on 20 reuse cycles, as reported by the returnable package supplier, which showed that R1 had a greater environmental impact than S1 for all the environmental impact categories except ozone depletion. Next, the sensitivity analysis was repeated using increasing reuses, including 30 and 40 cycles. The results confirmed that R1 generated more environmental impacts than S1, even if it was reused for 40 cycles. Figure S1 in the supporting information shows the sensitivity analysis based on the number of reuses of R1.

The second sensitivity analysis was conducted to analyze the sensitivity of the LCA results depending on the final consumer's location. The original LCA was conducted based on the assumption that the final consumer was in Vancouver, BC, Canada, and showed that R1 had a more extensive environmental impact for all the environmental impact categories except ozone depletion. The second sensitivity analysis was performed assuming that the final consumer was within a few km from brand owner A's location. The results showed that R1 generated more environmental impacts than S1, even if the final consumer was within 1 km from the brand owner. The returnable packaging system in case study 1 was the centralized model that the final consumer returned the used mailers to the returnable packaging supplier who cleaned and maintained the used mailers and shipped them to brand owner A. The long transport distance between the returnable packaging supplier in Salt Lake City, UT, USA and brand owner A in Anchorage, AK, USA (4754 km) was the primary reason why R1 still had a greater environmental impact than S1 with an extremely close final consumer location.

A follow-up sensitivity analysis was conducted based on the total trip distance of the package per cycle between the returnable packaging supplier, the brand owner, and the final consumer. In the original LCA scenario, the total trip distance of the package per cycle was 9836 km. The follow-up sensitivity analysis results showed that R1 had a lower environmental impact than S1 for 6 of the 10 environmental impact categories if the package's total trip distance was 1000 km. Based on global warming, one of the impact categories most affected by transport (Greenwood et al., 2021), the break-even trip distance where R1 and S1 had a similar environmental impact, also was approximately 1000 km per cycle. Figure S2 in the supporting information shows the results of the second and follow-up sensitivity analyses.

### Case study 2: Returnable box (R2) versus expendable box (S2)

The results of the life cycle impact assessment of the returnable box (R2) and expendable corrugated paperboard box (S2) are listed in Tables S4 and S5 in the supporting information, respectively.

**FIGURE 5:**
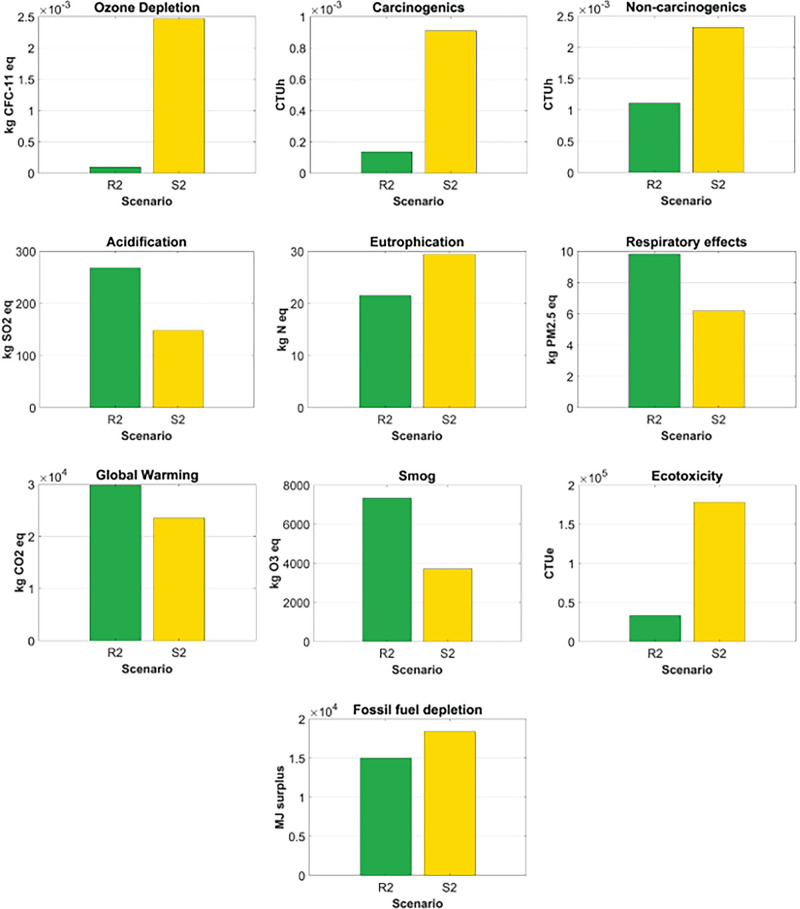
Comparative analysis of case study 2: R2 versus S2. Underlying data for this figure are available in Tables S5 and S6 of Supporting Information.

R2 had a lower environmental impact than S2 for 6 of the 10 environmental impact categories, including ozone depletion, eutrophication, carcinogenics, non-carcinogenics, ecotoxicity, and fossil fuel depletion. The most considerable difference was global warming, in which S2 generated over 25 times more environmental impact than R2. The second most significant difference was carcinogenics, where S2 had 576% more environmental impact than R2. The difference for ecotoxicity also was significant. For the environmental impact categories where R2 had a greater environmental impact than S2, the differences between the two packaging options were relatively smaller than those for the categories where R2 had less environmental impact than S2. The primary reasons that led to the difference in the environmental impacts between R2 and S2 were the heavier weight and the longer supply chains of R2. Figure 5 depicts the comparative analysis of R2 and S2.

**FIGURE 6:**
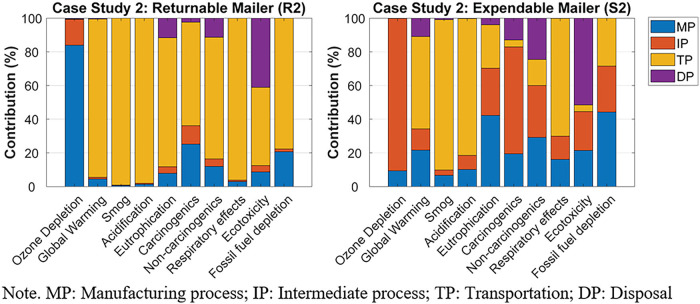
The normalized contribution analyses for case study 2's returnable mailer (R2) and expendable mailer (S2). Underlying data for this figure are available in Tables S5 and S6 of Supporting Information.

The normalized contribution of each life cycle stage's environmental impact, for R2 and S2, can be found in Figure 6. For R2, the greatest contribution to all the environmental impact categories, except ozone depletion, was the transportation phase. Specifically, the long-haul transport between brand owner B in Vancouver, BC, Canada, and the final consumer in Toronto, ON, Canada, and the crude oil production and diesel refinery process for the gas consumed during the transports were the primary contributors to 9 of the 10 environmental impact categories. Material production was the greatest contributor to ozone depletion due to the chlorine gas used for polyester resin production.

For S2, material production primarily contributed to fossil fuel depletion and eutrophication. Specifically, the natural gas used for corrugated paperboard production significantly contributed to fossil fuel depletion. During corrugated paperboard production, the spoil from coal mining was the greatest contributor to eutrophication. The intermediate process had the most significant contribution to ozone depletion, carcinogenics, and non-carcinogenics, mainly due to the organic solvent used for the printing process of the corrugated boxes. The transportation phase contributed the most to global warming, smog, acidification, and respiratory effects because of the long-haul transport between brand owner B and the final consumer, and the crude oil production and diesel refinery process for the gas consumed during the transports. The disposal phase, such as municipal incineration, was the largest contributor to eutrophication and ecotoxicity.

The first sensitivity analysis for case study 2 was performed to analyze how the overall LCA results were affected by the number of reuses of R2, specifically for a smaller number of reuses. The original scenario of the LCA was conducted based on the average number of reuses provided by brand owner B (40 cycles). The results showed that R2 had lower environmental impacts than S2 for 6 of the 10 environmental impact categories. The sensitivity analysis was conducted for 20, 15, 10, 5, and 2 cycles. The results showed that R2 still had lower environmental impacts than S2 for 6 of the 10 environmental impact categories if it was reused for 20 cycles. However, R2 had more environmental impacts than S2 for 6 of the 10 environmental impact categories when it was reused for 15 cycles. These findings can indicate that the exact number of returns to generate a favorable outcome, 60% less impactful than expendable, lies between 15 and 20 cycles. Figure S3 in the supporting information depicts the results of the first sensitivity analysis based on the number of reuses of R2.

The second sensitivity analysis for case study 2 was performed to analyze the sensitivity of the overall LCA results to the final consumer's locations in Canada, which was the primary uncertainty factor of the original LCA scenario. In the original scenario conducted in Toronto, ON, R2 had lower environmental impacts than S2 for 6 of the 10 categories. However, the LCA results changed depending on the trip distance between brand owner B and the final consumer's location. For this sensitivity analysis, six major cities across Canada were chosen, including Vancouver, Calgary, Regina, Winnipeg, Toronto, Montreal, and Halifax. Table 2 shows the total trip distance of the packages between brand owner B and the hypothetical final consumer locations.

**TABLE 2:** Changes in the packaging trip distances per cycle depending on the consumer location.

Consumer location	Total distance per cycle (km)
Vancouver, BC	32
Calgary, AB	1,942
Regina, SK	3,448
Winnipeg, MB	4,588
Toronto, ON (base)	8,414
Montreal, QB	9,112
Halifax, NS	12,330

The second sensitivity analysis results showed that R2 had a lower environmental impact than S2 for all the environmental impact categories when the final consumer was in Vancouver, BC. If the final consumer was in Calgary, AB, R2 had a lower environmental impact than S2 for 8 of the 10 environmental impact categories. When the consumer was in Regina, SK, or Winnipeg, MB, R2 generated less environmental burdens than S2 for 7 of the 10 impact categories. While R2 had a more negligible environmental impact than S2 for 6 of the 10 environmental impact categories when the consumer was in Toronto, ON, or Montreal, QB, R2 and S2 had a lower environmental impact for the same number of impact categories when the consumer was in Halifax, NS. Figure S4 in the supporting information shows the results of the second sensitivity analysis based on the final consumer location.

As global warming was one of the primary environmental impact categories affected most by different transport distances, a follow-up sensitivity analysis was conducted for global warming to find the break-even distance where R2 and S2 had similar environmental impacts. The two packaging options had similar environmental burdens for global warming at the total packaging trip distance of 2480 km. Thus, R2 had a lower environmental impact on global warming than S2 if the final consumer was within 1240 km from brand owner B in Vancouver.

## DISCUSSION

For case study 1, based on a centralized returnable packaging model with 20 return cycles, the comparative LCA found that the returnable mailer (R1) had greater impact than the expandable mailer (S1) in 9 of the 10 environmental impact categories. In particular, the impact of R1 was significantly higher in terms of smog (6 times) and acidification (4.8 times). The main factors leading to these results were the longer supply chain transport distance of R1 and the crude oil production and diesel refinery process behind the gas consumed during transports. In addition, sensitivity analyses revealed R1 to still generate a larger impact than S1 in 9 of the 10 environmental impact categories even if it was reused for 40 cycles or if the final consumer's location was within a few km of brand owner A's location. This was attributed to the long transport distance between brand owner A in Anchorage, AK, USA and the returnable packaging supplier in Salt Lake City, UT, USA (4754 km). With respect to global warming, which was one of the impact categories sensitive to transport, the break-even trip distance at which R1 and S1 had roughly the same environmental impact was 1000 km per cycle.

For case study 2, based on a decentralized returnable packaging model with 40 reuse cycles, the returnable box (R2) had a smaller impact than the expendable corrugated paperboard box (S2) in 6 of the 10 environmental impact categories. Those six were ozone depletion, eutrophication, carcinogenics, non-carcinogenics, ecotoxicity, and fossil fuel depletion; meanwhile, S2 had a smaller impact in terms of acidification, respiratory effects, global warming, and smog. For R2, the transportation phase was the largest contributor to most impact categories. For S2, impact categories differed in their primary contributors, which included corrugated paperboard production, spoil from coal mining, the organic solvent used in the printing process, and municipal incineration. Sensitivity analyses revealed that R2 had greater environmental impacts than S2 for 6 of the 10 environmental impact categories when it was reused for 15 cycles. However, the environmental impact of R2 became smaller than that of S2 in more than five environmental impact categories if the final consumer's location was one of Calgary, AB; Regina, SK; Winnipeg, MB; Toronto, ON; or Montreal, QB. When the final consumer was in Halifax, NS, the two packaging options were equal in terms of the number of categories in which each had smaller impact. With respect to global warming, the two packaging options had similar environmental impacts at a total trip distance of 2480 km.

The two case studies involved different business models as well as different returnable packaging management models. While brand owner A in case study 1 sold skirts to general consumers, brand owner B in case study 2 rented baby clothing for a certain period only to consumers who purchased its subscription-based memberships. With regard to returnable packaging management, brand owner A in case study 1 used the centralized model, where the final consumer returned the empty used package to returnable packaging supplier A in every shipping cycle. Returnable packaging supplier A then maintained the returned packages and supplied them again to brand owner A. In contrast, brand owner B in case study 2 used the decentralized model, directly managing and maintaining packages without support from returnable packaging supplier B. When consumers returned the rented baby clothing, they shipped it back to brand owner B using a returnable package they received from brand owner B.

The results of this work imply that the environmental performance of returnable packaging in the eCommerce supply chain is dependent on the total trip distance of the packaging and the number of reuses. Total trip distance is affected by the packaging management model (i.e., centralized vs. decentralized) and by the locations of the final consumer, brand owner, and returnable packaging supplier. As the decentralized model requires a shorter total trip distance than does the centralized model, it can be a better option if brand owners have the capability to maintain and manage returnable packages. For the centralized model, selecting optimized locations of packaging supplier maintenance facilities and depots is key to reducing environmental impact on account of those facilities directly affecting total trip distance. Packaging suppliers should optimize facility locations through a proper supply chain analysis based on the partner's (e.g., brand owner's) market and consumer data.

Meanwhile, the number of reuses is primarily affected by packaging durability and return rate. This study found that the subscription-based rental model in case study 2 featured a significantly higher return rate (100% package return) than the general selling model of case study 1 (70% package return). With these two cases, differences in business and returnable packaging management models might be the primary factors affecting packaging return rates. For one, the rental business model and the typical selling model had different levels of consumer engagement and return rates. In case study 1, if final consumers forgot to return or lost the packages, they could not be refunded the returnable packaging deposit (∼$3). Meanwhile, in case study 2, the final consumers had to return the products, not just packages, to avoid a non-return penalty (∼$80). In both cases, consumers used a returnable package they had already received from the brand owner. Thus, the higher potential financial loss that could occur due to failure of product return was the primary driver of the 100% packaging return in case study 2. As return rate is one of the primary factors determining the environmental impact of returnable packaging, the rental model with its higher return rate could be better suited for eCommerce business models employing a returnable packaging system.

## CONCLUSION

The results of this study indicate that the environmental burden of returnable packaging is primarily driven by its total trip distance and the number of times it is reused. For Canadian eCommerce, returnable packaging could have a competitive edge in terms of environmental sustainability if the brand owner uses the subscription-based rental business model and the returnable packaging is supplied and managed according to the decentralized model.

The findings from this study inform primary suggestions by which returnable packaging suppliers and brand owners can further reduce the environmental impacts of returnable packages. First, returnable packaging suppliers that use the centralized model need to consider establishing more maintenance depots in multiple regions within Canada to reduce total package trip distance. Second, they also need to enhance packaging durability to allow a greater number of reuses. Third, brand owners must review the total package trip distance before adopting a returnable packaging system. Finally, if brand owners have the capability to maintain and manage returnable packages on their own, they need to use the decentralized returnable packaging management system.

Future research could explore precise break-even points for both the distance travelled and the number of reuses required to break even for each of the 10 mid-point environmental categories. Additional research is required to understand how different eCommerce product categories with different mass, shape, and size perform.

This research could be further expanded to investigate consumer participation and behavioral tendencies regarding returnable mailer packaging. Understanding, the target number of reuses or the most efficient distances for the reusable eCommerce packaging will ensure it is deployed only in instances in which it reduces environmental impacts.

Furthermore, a cost analysis could be completed to understand the financial impacts of reusable and expendable mailers to provide business owners further clarity on the benefits of each packaging system.

## Supplementary Information

**Supporting Information S1**: This supporting information provides insights into two case studies comparing sustainable packaging options. Tables S1 and S2 show the life cycle inventory databases used for case study 1 returnable mailer and expendable mailer, and case study 2 returnable box and expendable box, respectively. Tables S3 and S4 show the results of the life cycle impact assessment of the returnable mailer and the expendable mailer analyzed in case study 1. Figures S1 and S2 show the results of sensitivity analysis based on the number of reuses of the returnable mailer, and the results of sensitivity analysis of the final consumer's location and the package's total trip distance per cycle in case study 1, respectively. Tables S5 and S6 show the results of the life cycle impact assessment of the returnable box and the expendable corrugated paperboard box analyzed in case study 2. Figures S3 and S4 show the results of sensitivity analysis based on the number of reuses of the returnable box, and the results of sensitivity analysis of the final consumer's location and the package's total trip distance per cycle in case study 2, respectively.

## Data Availability

The data that support the findings of this study are available from the corresponding author upon reasonable request.

## References

[CR1] Accorsi, R., Baruffaldi, G., & Manzini, R. (2020). A closed-loop packaging network design model to foster infinitely reusable and recyclable containers in food industry. *Sustainable Production and Consumption*, *24*, 48–61. 10.1016/J.SPC.2020.06.014

[CR2] Bare, J. (2014). Tool for the Reduction and Assessment of Chemical and Other Environmental Impacts (TRACI) TRACI version 2.1 user's guide. *U.S. EPA Office of Research and Development, EPA/600/R-12/554*.

[CR3] Baskoro, M. L. (2020). Exploring the emerging trends of reusable shipping packaging for e-commerce. *JISA (Jurnal Informatika Dan Sains)*, *3*(2), Article 2. 10.31326/jisa.v3i2.832

[CR4] Coelho, P. M., Corona, B., ten Klooster, R., & Worrell, E. (2020). Sustainability of reusable packaging–Current situation and trends. *Resources, Conservation & Recycling: X*, *6*, 100037. 10.1016/J.RCRX.2020.100037

[CR5] DuPont. (2017). Environmental product declaration DuPont™ TYVEK® mechanically fastened air and water barrier system. DePont. https://www.dupont.com/content/dam/dupont/amer/us/en/performance-building-solutions/public/documents/en/102-1_DuPont_EPD_Tyvek_Mechanically-Fastened.pdf

[CR6] Environment and Climate Change Canada. (2019). *Taking stock: Reducing food loss and waste in Canada*. https://www.canada.ca/content/dam/eccc/food-loss-and-waste/Taking%20Stock%20Report%20EN%20Final.pdf

[CR7] Escursell, S., Llorach-Massana, P., & Roncero, M. B. (2021). Sustainability in e-commerce packaging: A review. *Journal of Cleaner Production*, *280*, 124314. 10.1016/j.jclepro.2020.124314 32989345 10.1016/j.jclepro.2020.124314 PMC7511172

[CR8] Frischknecht, R., Jungbluth, N., Althaus, H.-J., Doka, G., Dones, R., Heck, T., Hellweg, S., Hischier, R., Nemecek, T., Rebitzer, G., & Spielmann, M. (2005). The ecoinvent database: Overview and methodological framework. *International Journal of Life Cycle Assessment*, *10*, 3–9.

[CR9] Goellner, K. N., & Sparrow, E. (2014). An environmental impact comparison of single-use and reusable thermally controlled shipping containers. *International Journal of Life Cycle Assessment*, *19*(3), 611–619. 10.1007/S11367-013-0668-Z/FIGURES/7

[CR10] Golding, A. (1999). *Reuse of Primary Packaging—Final Report*. Abfallberatung Müllvermeidung & Recyling.

[CR11] Greenwood, S. C., Walker, S., Baird, H. M., Parsons, R., Mehl, S., Webb, T. L., Slark, A. T., Ryan, A. J., & Rothman, R. H. (2021). Many happy returns: Combining insights from the environmental and behavioural sciences to understand what is required to make reusable packaging mainstream. *Sustainable Production and Consumption*, *27*, 1688–1702. 10.1016/J.SPC.2021.03.022

[CR12] Hugill, R., Ley, K., & Rademan, K. (2021). *The rise of reusable packaging: Understanding the impact and mapping a path to scale*. Fashion for Good. https://reports.fashionforgood.com/wp-content/uploads/2021/04/Reusable_Packaging_Report_April_2021.pdf

[CR14] Jestratijevic, I., Maystorovich, I., & Vrabič-Brodnjak, U. (2022). The 7 Rs sustainable packaging framework: Systematic review of sustainable packaging solutions in the apparel and footwear industry. *Sustainable Production and Consumption*, *30*, 331–340. 10.1016/J.SPC.2021.12.013

[CR15] Koskela, S., Dahlbo, H., Judl, J., Korhonen, M. R., & Niininen, M. (2014). Reusable plastic crate or recyclable cardboard box? A comparison of two delivery systems. *Journal of Cleaner Production*, *69*, 83–90. 10.1016/J.JCLEPRO.2014.01.045

[CR16] Lee, S. G., & Xu, X. (2004). A simplified life cycle assessment of re-usable and single-use bulk transit packaging. *Packaging Technology and Science*, *17*(2), 67–83. 10.1002/PTS.643

[CR17] Levi, M., Cortesi, S., Vezzoli, C., & Salvia, G. (2011). A comparative life cycle assessment of disposable and reusable packaging for the distribution of Italian fruit and vegetables. *Packaging Technology and Science*, *24*(7), 387–400. 10.1002/PTS.946

[CR18] Macarthur, E. (2013). Towards the circular economy. *Journal of Industrial Ecology*, *2*(1), 23–44.

[CR19] Mollenkopf, D., Closs, D., Twede, D., Lee, S., & Burgess, G. (2005). Assessing the viability of reusable packaging: A relative cost approach. *Journal of Business Logistics*, *26*(1), 169–197. 10.1002/J.2158-1592.2005.TB00198.X

[CR20] Soroka, W. (2007). *Illustrated glossary of packaging terminology*. DEStech Publications, Inc.

[CR22] Tua, C., Biganzoli, L., Grosso, M., & Rigamonti, L. (2019). Life cycle assessment of Reusable Plastic Crates (RPCs). *Resources 2019*, *8*(2), 110. 10.3390/RESOURCES8020110

[CR23] Twede, D., & Clarke, R. (2008). Supply chain issues in reusable packaging. *Journal of Marketing Channels*, *12*(1), 7–26. 10.1300/J049V12N01_02

[CR24] USEPA. (2020). *Advancing Sustainable Materials Management: 2018 Fact Sheet. United States Environmental Protection Agency (USEPA)*. https://www.epa.gov/sites/default/files/2021-01/documents/2018_ff_fact_sheet_dec_2020_fnl_508.pdf

[CR25] USLCI. (2012). *U.S. life cycle inventory database*. National Renewable Energy Laboratory. https://www.lcacommons.gov/nrel/search

[CR28] Zimmermann, T., & Bliklen, R. (2020). Single-use vs. reusable packaging in e-commerce: comparing carbon footprints and identifying break-even points. *GAIA - Ecological Perspectives for Science and Society*, *29*(3), 176–183. 10.14512/gaia.29.3.8

