# Supplementary material for: Environmental analysis of returnable packaging systems in different eCommerce business and packaging management models
Source: J Ind Ecol. 2024 Aug 13;28(6):1493–506. doi: 10.1111/jiec.13537 (PMC11667646; doi:10.1111/jiec.13537)

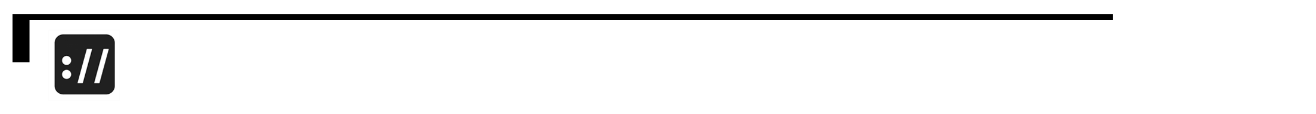


SUPPORTING INFORMATION FOR:

Park, J., Waqar, Z., Snyder, W.R. (2024). Environmental Analysis of Returnable Packaging Systems in Different eCommerce Business and Packaging Management Models. *Journal of Industrial Ecology.*


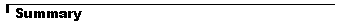


This supporting information provides insights into two case studies comparing sustainable packaging options. Tables S1 and S2 show the life cycle inventory databases used for case study 1 returnable mailer and expendable mailer, and case study 2 returnable box and expendable box, respectively. Tables S3 and S4 show the results of the life cycle impact assessment of the returnable mailer and the expendable mailer analyzed in case study 1. Figures S1 and S2 show the results of sensitivity analysis based on the number of reuses of the returnable mailer, and the results of sensitivity analysis of the final consumer’s location and the package’s total trip distance per cycle in case study 1, respectively. Tables S5 and S6 show the results of the life cycle impact assessment of the returnable box and the expendable corrugated paperboard box analyzed in case study 2. Figures S3 and S4 show the results of sensitivity analysis based on the number of reuses of the returnable box, and the results of sensitivity analysis of the final consumer’s location and the package’s total trip distance per cycle in case study 2, respectively.


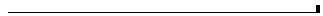


**Table S1.** Life cycle inventory of the returnable mailer (R1) and the expandable mailer (S1) based on the functional unit in case study 1

| **Packaging Format** | **Components** | **Materials** | **Inventory name** | **Values/functional unit** | **Inventory** | **Pedigree Score** |
| --- | --- | --- | --- | --- | --- | --- |
| Returnable Mailer (R1) | Shipping Label | Paper | Kraft paper, bleached, at plant/US-US EI U | 16.4 kg | US-EI 2.2 | (1,2,1,2,2,5) |
|  | ID Label | Silicon | Silicon, multi-si, casted, at plant/US - US - E1 U | 0.07 kg | US-EI 2.2 | (2,3,1,3,2,5) |
|  | Mailer Bag | PP | Polypropylene, granulate, at plant/US-US EI U;  Extrusion, plastic film, at plant/US-US EI U;  Weaving, synthetic yarn, at plant/US-US EI U | 73.45 kg | US-EI 2.2 | (1,3,1,2,2,3)  (2,3,1,3,2,5)  (2,3,1,3,2,5) |
|  | Straps | Nylon 6 | Nylon 6, at plant/ US US-EI U | 11.39 kg | US-EI 2.2 | (1,3,1,3,2,5) |
|  | Spray cleaner | | Alkaline cleaners, CIP | 0.25 kg | US-EI 2.2 | (2,3,1,3,2,5) |
|  | Detergent | | Alkaline detergents, CIP | 0.18 kg | US-EI 2.2 | (2,3,1,3,2,5) |
|  | Transportation | | Transport, ocean freighter, diesel powered, US/tkm/RNA | 2360.40 tkm | US LCI | (1,3,1,1,2,3) |
|  |  |  | Transport, single unit truck, long haul, diesel powered,West/tkm/RNA | 69.65 tkm | US LCI | (1,3,1,1,2,3) |
|  |  |  | Transport, single unit truck, long haul, diesel powered, Southwest/tkm/RNA | 314.43 tkm | US LCI | (1,3,1,1,2,3) |
|  |  |  | Transport, single unit truck, long haul, diesel powered, Alaska/tkm/RNA | 233.34 tkm | US LCI | (1,3,1,1,2,3) |
|  |  |  | Transport, single unit truck, long haul, diesel powered/tkm/RNA | 102.78 tkm | US LCI | (1,3,1,1,2,3) |
|  |  |  | Transport, single unit truck, long haul, diesel powered, Southwest/tkm/RNA | 314.43 tkm | US LCI | (1,3,1,1,2,3) |
| Expandable Mailer (S1) | Shipping Label | Paper | Kraft paper, bleached, at plant/US-US EI U | 16.4 kg | US-EI 2.2 | (1,2,1,2,2,5) |
|  | Mailer Bag | HDPE | High density polyethylene, granulate, at plant/US- US-EI U  Spinning fibre, synthetic/US U- US-EI U | 7.85 kg | US-EI 2.2 | (2,3,2,2,2,5) (2,3,1,3,2,5) |
|  | Transportation | | Transport, single unit truck, long haul, diesel powered, Southeast/tkm/RNA | 1208.25 tkm | US LCI | (1,3,1,1,2,3) |
|  |  |  | Transport, single unit truck, short haul, diesel powered, Alaska/tkm/RNA | 2.60 tkm | US LCI | (1,3,1,1,2,3) |
|  |  |  | Transport, single unit truck, long haul, diesel powered/tkm/RNA | 611.76 tkm | US LCI | (1,3,1,1,2,3) |

**Table S2.** Life cycle inventory of the returnable box (R2) and the expandable box (S2) based on the functional unit in case study 2

| **Packaging Format** | **Components** | **Materials** | **Inventory name** | **Values/functional unit** | **Inventory** | **Pedigree Score** |
| --- | --- | --- | --- | --- | --- | --- |
| Returnable Box (R2) | Shipping Label | Paper | Kraft paper, bleached, at plant/US US-EI U | 0.41 kg | US-EI 2.2 | (1,2,1,2,2,5) |
|  | ID Label | Silicon | Silicon, multi-si, casted, at plant/US US-EI U | 0.63 kg | US-EI 2.2 | (2,3,1,3,2,5) |
|  | Box walls | PP | Polypropylene, granulate, at plant/US-US EI U  Extrusion, plastic film, at plant/US US-EI U | 112.50 kg | US-EI 2.2  US-EI 2.2 | (1,3,1,2,2,3)  (2,3,1,3,2,5) |
|  |  | Polyester | Polyester resin, unsaturated, at plant/US- US-EI U  Spinning, synthetic fibres, at plant/US US-EI U | 107.50 kg | US-EI 2.2 | (1,3,1,3,2,5)  (2,3,1,3,2,5) |
|  | Zippers | Nylon 6 | Nylon 6, at plant/US-US EI U | 21.38 kg | US-EI 2.2 | (1,3,1,3,2,5) |
|  |  | Steel | Steel organic coated/GLO | 1.60 kg | Ecoinvent v3 | (2,3,1,3,2,5) |
|  |  | Nylon 6 | Nylon 6, at plant/ US US-EI U | 7.26 kg | US-EI 2.2 | (1,3,1,3,2,5) |
|  | D-rings | PP | Polypropylene, granulate, at plant/US-US EI U  Injection moulding {RoW}\| processing \| APOS, S | 0.03 kg | US-EI 2.2  Ecoinvent v3 | (1,3,1,2,2,3)  (2,3,1,3,2,5) |
|  | Detergent | | Alkaline detergents, CIP | 0.013 kg | US-EI 2.2 | (2,3,1,3,2,5) |
|  | Transportation | | Transport, ocean freighter, diesel powered/US | 9409.32 tkm | U.S. LCI | (1,3,1,1,2,3) |
|  |  |  | Transport, single unit truck, short haul, diesel powered/tkm/RNA | 4.00 tkm | U.S. LCI | (1,3,1,1,2,3) |
|  |  |  | Transport, single unit truck, short haul, diesel powered/tkm/RNA | 0.20 tkm | U.S. LCI | (1,3,1,1,2,3) |
|  |  |  | Transport, single unit truck, long haul, diesel powered/tkm/RNA | 1037.83 tkm | U.S. LCI | (1,3,1,1,2,3) |
|  |  |  | Transport, single unit truck, long haul, diesel powered/tkm/RNA | 1037.83 tkm | U.S. LCI | (1,3,1,1,2,3) |
| Expandable Box (S2) | Shipping Label | Paper | Kraft paper, bleached, at plant/US-US EI U | 16.40 kg | US-EI 2.2 | (1,2,1,2,2,5) |
|  | Box | Corrugated Paperboard | Corrugated board, recycled fibre, double wall, at plant/US- US EI U | 4500 kg | US-EI | (1,3,1,3,2,5) |
|  | Box Tape | PVC | Polyvinyl chloride resin, at plant/RNA | 51.60 kg | U.S LCI | (2,3,1,3,2,5) |
|  | Transportation | | Transport, single unit truck, short haul, diesel powered/tkm/RNA | 41.11 tkm | U.S. LCI | (1,3,1,1,2,3) |
|  |  |  | Transport, single unit truck, short haul, diesel powered/tkm/RNA | 68.52 tkm | U.S. LCI | (1,3,1,1,2,3) |
|  |  |  | Transport, single unit truck, long haul, diesel powered/tkm/RNA | 19217.58 tkm | U.S. LCI | (1,3,1,1,2,3) |
|  |  |  | Transport, single unit truck, long haul, diesel powered/tkm/RNA | 19217.58 tkm | U.S. LCI | (1,3,1,1,2,3) |

**Table S3.** The results of the life cycle impact assessment of the returnable mailer (R1) analyzed in case study 1. This data is used to create Figure 3 in manuscript.

| **Impact**  **Category** | **Unit** | **Total** | **MP** | **IP** | **TP** | **DP** |
| --- | --- | --- | --- | --- | --- | --- |
| Ozone Depletion | kg CFC-11 eq | 1.93E-05 | 5.83E-07 | 1.84E-05 | 1.92E-07 | 1.44E-07 |
| Global Warming | kg CO_2_ eq | 5.86E+03 | 2.58E+02 | 4.18E+02 | 5.16E+03 | 1.91E+01 |
| Smog | kg O_3_ eq | 1.38E+03 | 1.17E+01 | 1.83E+01 | 1.35E+03 | 2.73E-01 |
| Acidification | kg SO_2_ eq | 5.59E+01 | 8.22E-01 | 2.12E+00 | 5.29E+01 | 9.79E-03 |
| Eutrophication | kg N eq | 5.61E+00 | 1.29E-01 | 1.23E+00 | 3.16E+00 | 1.09E+00 |
| Carcinogenics | CTUh | 9.64E-05 | 6.50E-06 | 2.07E-05 | 6.89E-05 | 3.33E-07 |
| Non-carcinogenics | CTUh | 7.84E-04 | 3.69E-06 | 6.97E-05 | 6.64E-04 | 4.62E-05 |
| Respiratory effects | kg PM2.5 eq | 1.90E+00 | 6.13E-02 | 1.27E-01 | 1.71E+00 | 1.25E-03 |
| Ecotoxicity | CTUe | 2.12E+04 | 3.77E+02 | 1.71E+03 | 1.28E+04 | 6.23E+03 |
| Fossil fuel depletion | MJ surplus | 1.09E+04 | 9.28E+02 | 3.00E+02 | 9.66E+03 | 2.07E+00 |

Note. MP: Manufacturing process; IP: Intermediate process; TP: Transportation; DP: Disposal

**Table S4.** The results of the life cycle impact assessment of the expendable mailer (S1) analyzed in case study 1. This data is used to create Figure 3 in manuscript.

| **Impact**  **Category** | **Unit** | **Total** | **MP** | **IP** | **TP** | **DP** |
| --- | --- | --- | --- | --- | --- | --- |
| Ozone Depletion | kg CFC-11 eq | 1.96E-05 | 2.35E-06 | 1.70E-05 | 2.87E-08 | 1.79E-07 |
| Global Warming | kg CO_2_ eq | 1.46E+03 | 3.39E+02 | 3.38E+02 | 6.85E+02 | 1.02E+02 |
| Smog | kg O_3_ eq | 1.97E+02 | 1.54E+01 | 1.71E+01 | 1.64E+02 | 6.35E-01 |
| Acidification | kg SO_2_ eq | 9.61E+00 | 1.19E+00 | 1.79E+00 | 6.61E+00 | 1.75E-02 |
| Eutrophication | kg N eq | 3.18E+00 | 1.70E-01 | 1.01E+00 | 3.90E-01 | 1.61E+00 |
| Carcinogenics | CTUh | 3.98E-05 | 1.15E-05 | 1.72E-05 | 1.03E-05 | 8.67E-07 |
| Non-carcinogenics | CTUh | 2.34E-04 | 9.92E-06 | 5.80E-05 | 9.92E-05 | 6.68E-05 |
| Respiratory effects | kg PM2.5 eq | 4.29E-01 | 9.77E-02 | 1.06E-01 | 2.24E-01 | 1.67E-03 |
| Ecotoxicity | CTUe | 1.46E+04 | 6.90E+02 | 1.38E+03 | 1.92E+03 | 1.06E+04 |
| Fossil fuel depletion | MJ surplus | 3.35E+03 | 1.66E+03 | 2.40E+02 | 1.44E+03 | 2.56E+00 |

Note. MP: Manufacturing process; IP: Intermediate process; TP: Transportation; DP: Disposal

**Figure S1.** Sensitivity analysis based on the number of reuses of the returnable mailer analyzed in case study 1 (R1)


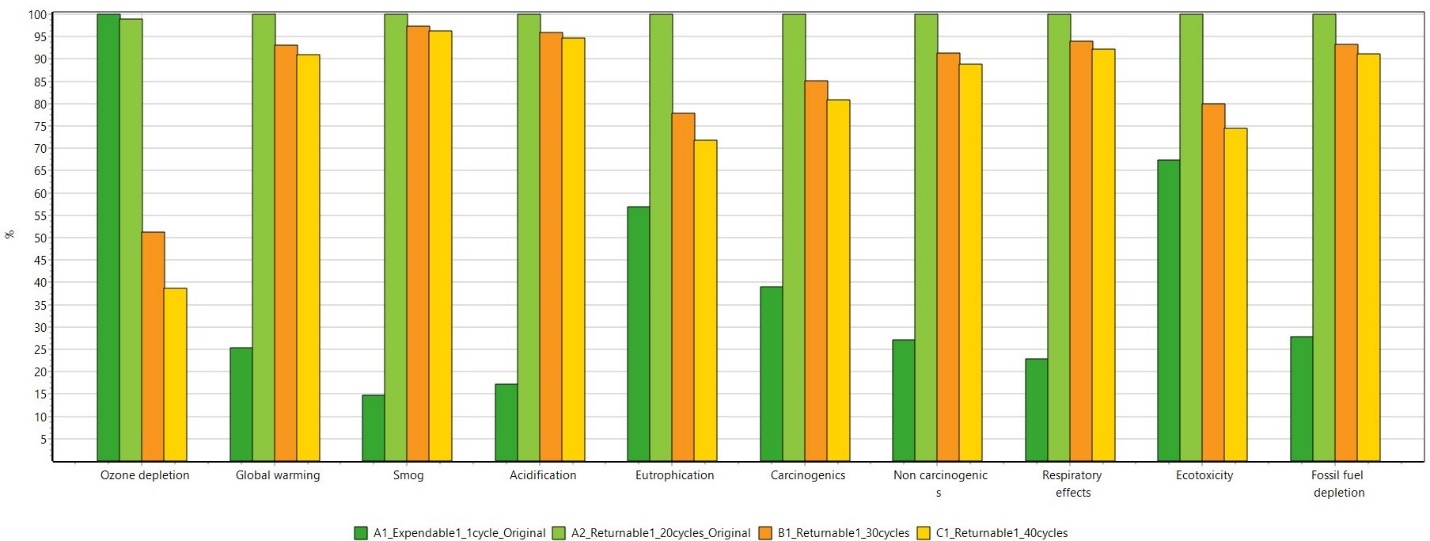


**Figure S2.** Sensitivity analysis of the final consumer’s location and the package’s total trip distance per cycle in case study 1


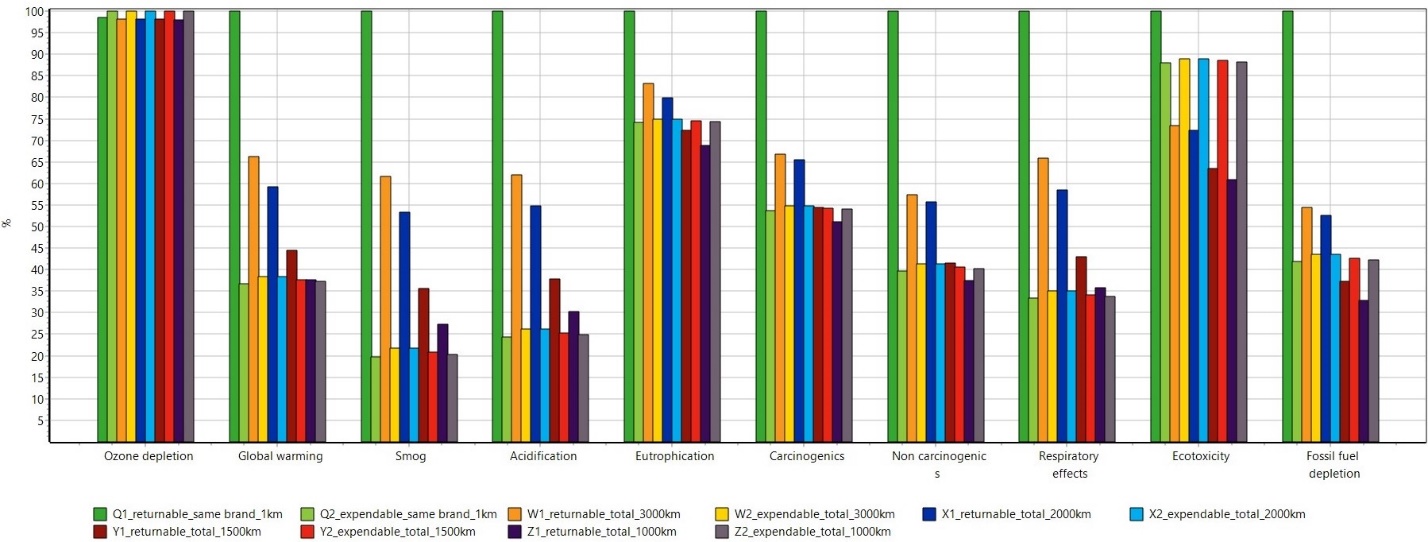


**Table S5.** The results of the life cycle impact assessment of the returnable box (R1) analyzed in case study 2. This data is used to create Figure 4 in manuscript.

| **Impact**  **Category** | **Unit** | **Total** | **MP** | **IP** | **TP** | **DP** |
| --- | --- | --- | --- | --- | --- | --- |
| Ozone Depletion | kg CFC-11 eq | 9.42E-05 | 7.91E-05 | 1.44E-05 | 2.32E-07 | 4.30E-07 |
| Global Warming | kg CO_2_ eq | 2.98E+04 | 1.33E+03 | 3.03E+02 | 2.80E+04 | 1.48E+02 |
| Smog | kg O_3_ eq | 7.33E+03 | 4.56E+01 | 1.43E+01 | 7.27E+03 | 1.24E+00 |
| Acidification | kg SO_2_ eq | 2.68E+02 | 3.66E+00 | 1.50E+00 | 2.63E+02 | 4.13E-02 |
| Eutrophication | kg N eq | 2.15E+01 | 1.69E+00 | 8.36E-01 | 1.65E+01 | 2.50E+00 |
| Carcinogenics | CTUh | 1.35E-04 | 3.39E-05 | 1.48E-05 | 8.29E-05 | 3.14E-06 |
| Non-carcinogenics | CTUh | 1.11E-03 | 1.32E-04 | 4.91E-05 | 7.99E-04 | 1.25E-04 |
| Respiratory effects | kg PM2.5 eq | 9.82E+00 | 2.86E-01 | 9.13E-02 | 9.43E+00 | 3.62E-03 |
| Ecotoxicity | CTUe | 3.32E+04 | 2.87E+03 | 1.24E+03 | 1.54E+04 | 1.36E+04 |
| Fossil fuel depletion | MJ surplus | 1.50E+04 | 3.09E+03 | 2.47E+02 | 1.16E+04 | 6.39E+00 |

Note. MP: Manufacturing process; IP: Intermediate process; TP: Transportation; DP: Disposal

**Table S6.** The results of the life cycle impact assessment of the expendable corrugated paperboard box (S2) analyzed in case study 2. This data is used to create Figure 4 in manuscript.

| **Impact**  **Category** | **Unit** | **Total** | **MP** | **IP** | **TP** | **DP** |
| --- | --- | --- | --- | --- | --- | --- |
| Ozone Depletion | kg CFC-11 eq | 2.47E-03 | 2.31E-04 | 2.24E-03 | 1.04E-07 | 4.50E-06 |
| Global Warming | kg CO_2_ eq | 2.35E+04 | 5.08E+03 | 2.97E+03 | 1.29E+04 | 2.56E+03 |
| Smog | kg O_3_ eq | 3.72E+03 | 2.48E+02 | 1.17E+02 | 3.32E+03 | 3.32E+01 |
| Acidification | kg SO_2_ eq | 1.48E+02 | 1.51E+01 | 1.24E+01 | 1.21E+02 | 2.93E-01 |
| Eutrophication | kg N eq | 2.94E+01 | 1.24E+01 | 8.23E+00 | 7.56E+00 | 1.15E+00 |
| Carcinogenics | CTUh | 9.11E-04 | 1.77E-04 | 5.79E-04 | 3.74E-05 | 1.18E-04 |
| Non-carcinogenics | CTUh | 2.32E-03 | 6.78E-04 | 7.14E-04 | 3.60E-04 | 5.68E-04 |
| Respiratory effects | kg PM2.5 eq | 6.21E+00 | 1.00E+00 | 8.62E-01 | 4.34E+00 | 8.66E-03 |
| Ecotoxicity | CTUe | 1.78E+05 | 3.82E+04 | 4.12E+04 | 6.97E+03 | 9.19E+04 |
| Fossil fuel depletion | MJ surplus | 1.84E+04 | 8.14E+03 | 5.03E+03 | 5.24E+03 | -6.51E+00 |

Note. MP: Manufacturing process; IP: Intermediate process; TP: Transportation; DP: Disposal

**Figure S3.** Results of the sensitivity analysis based on the number of reuses of the returnable box (R2) analyzed in case study 2


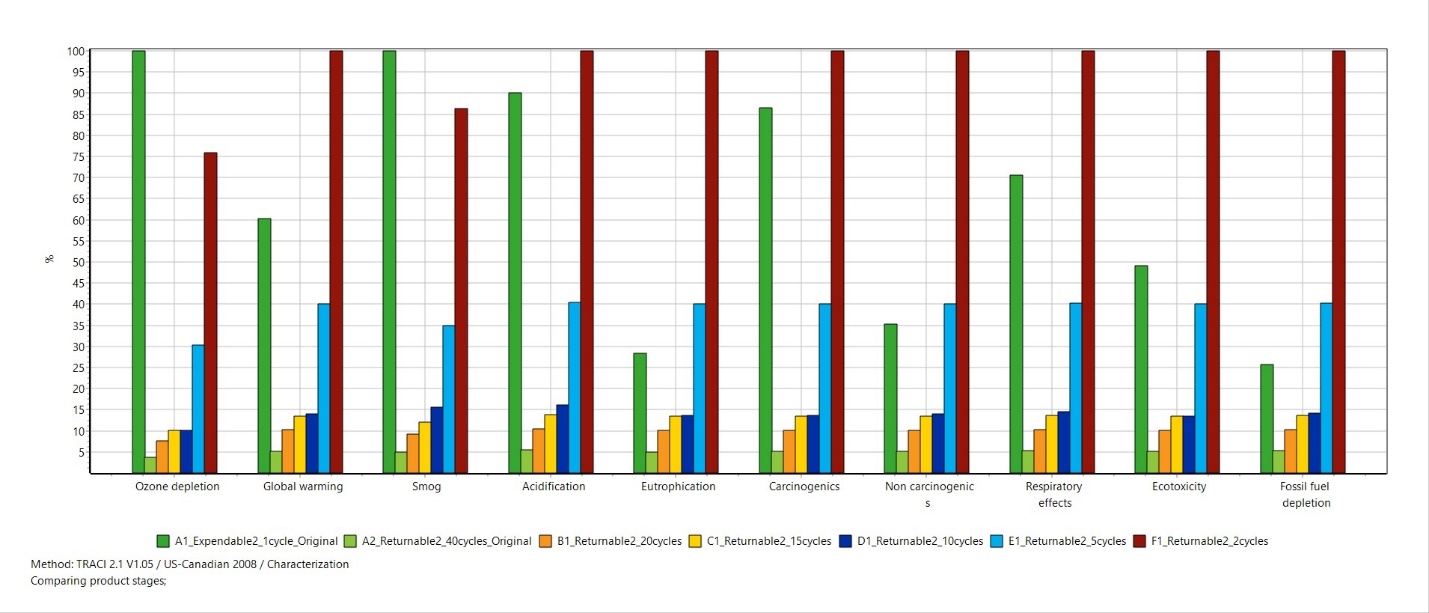


**Figure S4.** Results of the sensitivity analysis based on the final consumer location in case study 2


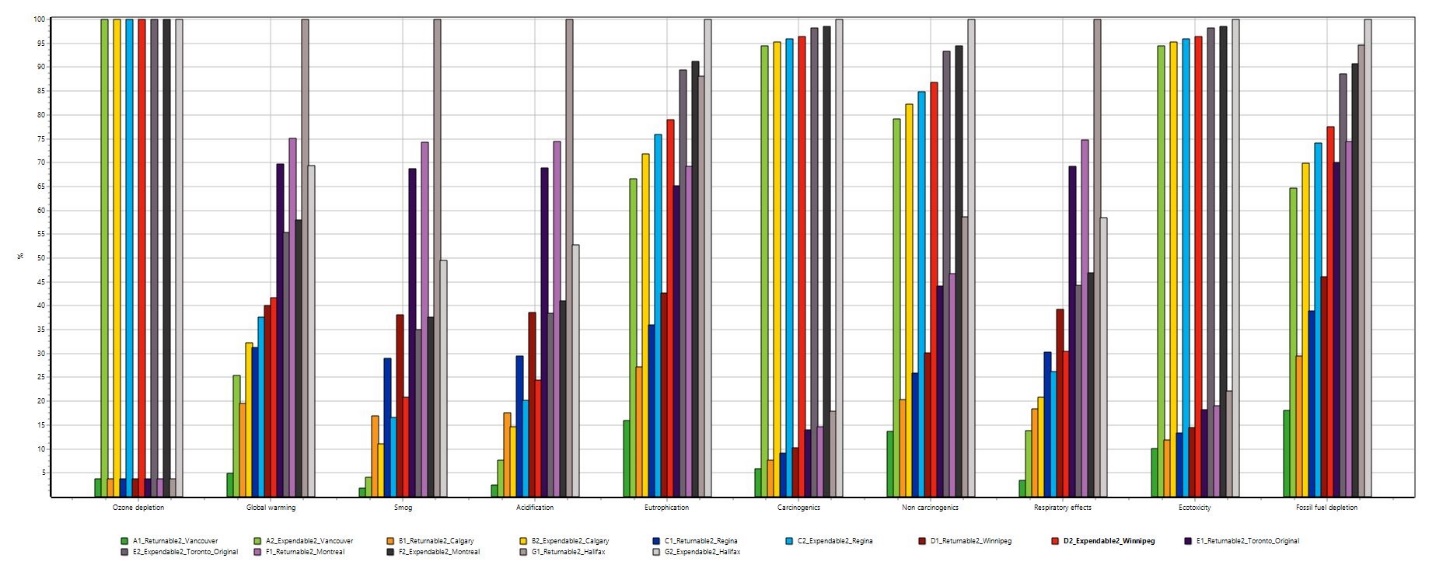

Supplement: Supplementary file 1 — Supporting Information S1: This supporting information provides insights into two case studies comparing sustainable packaging options. Tables S1 and S2 show the life cycle inventory databases used for case study 1 returnable mailer and expendable mailer, and case study 2 returnable box and expendable box, respectively. Tables S3 and S4 show the results of the life cycle impact assessment of the returnable mailer and the expendable mailer analyzed in case study 1. Figures S1 and S2 show the results of sensitivity analysis based on the number of reuses of the returnable mailer, and the results of sensitivity analysis of the final consumer's location and the package's total trip distance per cycle in case study 1, respectively. Tables S5 and S6 show the results of the life cycle impact assessment of the returnable box and the expendable corrugated paperboard box analyzed in case study 2. Figures S3 and S4 show the results of sensitivity analysis based on the number of reuses of the returnable box, and the results of sensitivity analysis of the final consumer's location and the package's total trip distance per cycle in case study 2, respectively. [file 44498_2024_2806013_MOESM1_ESM.docx]
